# Proteomics-Based Characterization of the Humoral Immune Response in Sporotrichosis: Toward Discovery of Potential Diagnostic and Vaccine Antigens

**DOI:** 10.1371/journal.pntd.0004016

**Published:** 2015-08-25

**Authors:** Anderson Messias Rodrigues, Geisa Ferreira Fernandes, Leticia Mendes Araujo, Paula Portella Della Terra, Priscila Oliveira dos Santos, Sandro Antonio Pereira, Tânia Maria Pacheco Schubach, Eva Burger, Leila Maria Lopes-Bezerra, Zoilo Pires de Camargo

**Affiliations:** 1 Department of Microbiology, Immunology and Parasitology, Discipline of Cellular Biology, Federal University of São Paulo, São Paulo, Brazil; 2 Instituto Nacional de Infectologia Evandro Chagas, Fundação Oswaldo Cruz (INI/Fiocruz), Rio de Janeiro, Brazil; 3 Department of Microbiology and Immunology, Biomedical Sciences Institute, Federal University of Alfenas, Alfenas, Brazil; 4 Instituto de Biologia (IBRAG), Universidade do Estado do Rio de Janeiro, Rio de Janeiro, Brazil; University of California San Diego School of Medicine, UNITED STATES

## Abstract

**Background:**

*Sporothrix schenckii* and associated species are agents of human and animal sporotrichosis that cause large sapronoses and zoonoses worldwide. Epidemiological surveillance has highlighted an overwhelming occurrence of the highly pathogenic fungus *Sporothrix brasiliensis* during feline outbreaks, leading to massive transmissions to humans. Early diagnosis of feline sporotrichosis by demonstrating the presence of a surrogate marker of infection can have a key role for selecting appropriate disease control measures and minimizing zoonotic transmission to humans.

**Methodology:**

We explored the presence and diversity of serum antibodies (IgG) specific against *Sporothrix* antigens in cats with sporotrichosis and evaluated the utility of these antibodies for serodiagnosis. Antigen profiling included protein extracts from the closest known relatives *S*. *brasiliensis* and *S*. *schenckii*. Enzyme-linked immunosorbent assays and immunoblotting enabled us to characterize the major antigens of feline sporotrichosis from sera from cats with sporotrichosis (n = 49), healthy cats (n = 19), and cats with other diseases (n = 20).

**Principal Findings:**

Enzyme-linked immunosorbent assay-based quantitation of anti-*Sporothrix* IgG exhibited high sensitivity and specificity in cats with sporotrichosis (area under the curve, 1.0; 95% confidence interval, 0.94–1; *P*<0.0001) versus controls. The two sets of *Sporothrix* antigens were remarkably cross-reactive, supporting the hypothesis that antigenic epitopes may be conserved among closely related agents. One-dimensional immunoblotting indicated that 3-carboxymuconate cyclase (a 60-kDa protein in *S*. *brasiliensis* and a 70-kDa protein in *S*. *schenckii*) is the immunodominant antigen in feline sporotrichosis. Two-dimensional immunoblotting revealed six IgG-reactive isoforms of gp60 in the *S*. *brasiliensis* proteome, similar to the humoral response found in human sporotrichosis.

**Conclusions:**

A convergent IgG-response in various hosts (mice, cats, and humans) has important implications for our understanding of the coevolution of *Sporothrix* and its warm-blooded hosts. We propose that 3-carboxymuconate cyclase has potential for the serological diagnosis of sporotrichosis and as target for the development of an effective multi-species vaccine against sporotrichosis in animals and humans.

## Author Summary

Sporotrichosis is a neglected fungal disease of humans and animals that remains a serious public-health problem. *Sporothrix* infections persist in cats, leading to continued transmission via cat-to-cat and cat-to-human contact. Cats are the major source of transmission of *Sporothrix brasiliensis* to the human population. We stress the importance of implementing health policies aimed at detecting *Sporothrix* infection in cats as an attempt to reduce massive zoonotic transmission to humans. Early diagnosis of feline sporotrichosis is critical to recognize outbreaks areas and effectively tackle future spread of the disease among humans. We explored the diversity of molecules that are expressed by *S*. *brasiliensis* and *S*. *schenckii* and that are recognized by immunoglobulin G. Upon infection, the cat delivers an IgG-mediated response against *Sporothrix* antigens, similar to the response in murine and human sporotrichosis. We detected remarkable cross-reactivity among *S*. *brasiliensis* and *S*. *schenckii* antigens, supporting the hypothesis that antigenic epitopes may be conserved among closely related species. One protein, 3-carboxymuconate cyclase, was prominent in immune profiles from infected animals, using both types of *Sporothrix* antigens. Knowledge of the immune response in feline sporotrichosis is critical to advancing techniques for serological diagnosis, developing vaccines, and improving our understanding of *Sporothrix* evolution.

## Introduction

*Sporothrix schenckii* was originally described in 1898 as the causal agent of a subcutaneous disease in humans in the Mid-Atlantic USA [1]. Subsequently, the disease was reported in rats naturally infected in southeastern Brazil [2] and later in a wide range of animals including dogs, cats, horses, cows, camels, dolphins, goats, mules, birds, pigs, and armadillos. Several *Sporothrix* spp., previously reported to be closely related to *S*. *schenckii*, are known to establish infections in various hosts, with dissimilar virulence traits [3, 4] and responsiveness to antifungal treatment [5].

The *S*. *schenckii* complex consists of at least four closely-related species [6, 7], ranging from geographically restricted agents such as *S*. *brasiliensis* [8, 9] to cosmopolitan pathogens such as *S*. *schenckii s*. *str*. and *S*. *globosa* [7, 10, 11]. *Sporothrix* spp. are endowed with an extraordinary ecological diversity [12–15]; they are frequently recovered from soil, plants debris, and insects (Coleoptera: Scolytidae). Phylogenetic data support a recent habitat shift within *Sporothrix* from plants to cats [9] that culminated in the largest epizootic transmission in southeastern Brazil [16–19]. Feline sporotrichosis emerged in the 1990s, with *S*. *brasiliensis* recovered from many outbreaks [8, 20]. More recently, *S*. *brasiliensis* has been recognized as a threat to humans [21–23] due to the massive zoonotic transmission in southeastern Brazil that affects thousands of patients regardless of whether they are immunocompetent or immunocompromised [9, 24–26].

Cats have been a source of *Sporothrix* spp. infection transmitted to humans and other animals [18, 19, 27]. Most human cases occurred in housewives and professionals who had contact with infected animals and a history of scratches or bites [21, 28]. The largest epidemic of sporotrichosis due to zoonotic transmission was reported in the State of Rio de Janeiro, Brazil [18, 19, 21, 23, 28]; since then, the incidence of sporotrichosis among animals, particularly cats, has increased [8, 28, 29]. More than 4,000 humans and 4,124 cats were diagnosed at Instituto Nacional de Infectologia (INI) Evandro Chagas /Fundação Oswaldo Cruz by 2012 [30]. Pereira et al. [29] observed that the majority of cats with sporotrichosis in Rio de Janeiro between 2005 and 2011 were male, mongrel, and unneutered, had a median age of 24 months, and presented with three or more cutaneous lesions in non-adjacent locations. This mycosis in cats is hard to treat and generally requires a long period of daily care; these animals do not always respond well to antifungal treatment [30].

*Sporothrix* is widely distributed in nature, and traumatic inoculation of plant organic matter is classically associated with infection [31]. Feline habits render cats more susceptible to the fungal agent because they are constantly in contact with soil, plant debris, and other cats that may be infected. In cats, a broad spectrum of clinical presentation ranges from single lesions to fatal systemic forms. After monitoring the feline epidemic for 4 years, Schubach et al. [18] observed that the lymphocutaneous form occurred less frequently than did involvement of the mucous membranes of the respiratory tract and upper digestive tract and multiple cutaneous lesions. Some animals present involvement of skin and various internal organs [32].

Cats are susceptible to a variety of fungal infections, including blastomycosis [33], histoplasmosis [34], cryptococcosis [35], candidiasis [36], dermatophytosis [37], aspergillosis [38], coccidioidomycosis [39], and sporotrichosis. Misdiagnosis results in ineffective treatment, which further worsens outcome. Major contributors toward misdiagnosis include the small number of affordable and effective treatment techniques as well as other social issues. Definitive diagnosis of feline sporotrichosis is based on mycological culture, micromorphological characterization, and mold-to-yeast conversion. Histopathological methods and cytopathological examination are useful tools for the presumptive diagnosis of *Sporothrix* infection in cats [40]. Detection of specific anti-*Sporothrix* antibodies offers a rapid alternative for accurate diagnosis [41–43]. We recently proposed a serological approach that employs an enzyme-linked immunosorbent assay (ELISA) to diagnose feline sporotrichosis [44] using purified antigen (the *S*. *schenckii* ConA binding fraction) and crude antigen, with high sensitivity and specificity.

There is a lack of information about feline sporotrichosis and the antigenic components involved in infection; therefore, the present study aimed to explore the diversity of molecules expressed by closely related species (*S*. *brasiliensis* and *S*. *schenckii*) and that are recognized by immunoglobulin G (IgG) in sera from cats naturally infected with *S*. *brasiliensis*. We found remarkable cross-reactivity among *S*. *brasiliensis* and *S*. *schenckii* antigens, and we identified an immunodominant molecule that is an important biomarker in feline sporotrichosis, irrespective of clinical manifestation. Here, we show that, although *S*. *brasiliensis* and *S*. *schenckii* may infect different warm-blooded hosts, infection result in highly similar IgG-mediated response in cats compared to humans, what is important for serodiagnosis and to the development of prophylactic or therapeutic vaccine against the enormous health burden of sporotrichosis in endemic areas. This knowledge may enable selection of potential biomarkers that can be used in seroepidemiological studies, diagnosis, and vaccine development, and may contribute to understanding of the pathogenesis of this infection in cats and humans.

## Methods

### Ethical approval

This study was performed in strict accordance with recommendations in the Guide for the Care and Use of Laboratory Animals of the National Institutes of Health. The protocol was approved by the Ethics in Research Committee of the Fundação Oswaldo Cruz, Rio de Janeiro, Brazil, under license number L-041/06. This study was also approved by the Institutional Ethics in Research Committee of the Federal University of São Paulo under protocol number 0244/11.

### Fungal strains and culture conditions

*S*. *brasiliensis* and *S*. *schenckii s*. *str*. from cats and humans were used for protein extraction (Table 1). The dimorphic nature of *Sporothrix* spp. was demonstrated by converting the fungus to its yeast form at 36°C in brain-heart infusion medium (Difco) and observing typical oval multibudding yeast cells. Molecular identification was performed and confirmed via DNA sequencing of the gene encoding calmodulin [25].

**Table 1:** *Sporothrix* isolates used in this study.

Strain	Other code	Species	Source	Origin	*CAL* Genbank	Reference
CBS 132990	Ss54	*S*. *brasiliensis*	Feline	Brazil	JQ041903	[9, 25, 45]
CBS 132021	5110	*S*. *brasiliensis*	Feline	Brazil	JF313351	[46]
CBS 132974	Ss118	*S*. *schenckii*	Human	Brazil	JX077126	[9, 25, 45]
CBS 132984	1099–18	*S*. *schenckii*	Human	USA	JF313360	[46]

CBS: Centraalbureau voor Schimmelcultures, Utrecht, The Netherlands; All “Ss” strains belong to the culture collection of Federal University of São Paulo, São Paulo, Brazil.

Isolates were selected because they had been previously characterized at the molecular level [3, 9, 45, 46]; crude exoantigen (CBS 132974 = Ss118; *S*. *schenckii s*. *str*.) was successfully used to diagnose feline sporotrichosis via ELISA [44]. *Sporothrix* spp. was grown on Sabouraud medium (Difco) agar slants at room temperature (20–25°C) for 7 days. Approximately 2x10^6^ conidia (≥85% viable cells) were used to inoculate 500-mL flasks containing 150 mL of brain-heart infusion broth. Cultures were incubated at 36°C in a rotary shaker (Multitron II, Infors HT) with constant orbital agitation at 110 rpm for 7 days.

### Whole cellular extracts

Whole extracts of *Sporothrix* yeast cells were obtained as described elsewhere [47]. Briefly, yeast cells (4 mL of each culture) were collected via centrifugation at 5000 x *g* for 10 min at 4°C and washed three times in ultrapure water. Pellets were frozen in liquid nitrogen and disrupted by gridding. Cells were macerated with a pestle until a fine powder was obtained. This cellular powder was vortexed for 30 min at 4°C in Tris-Ca^2+^ buffer (20 mM Tris-HCl pH 8.8, 2 mM CaCl_2_) containing a commercial cocktail of protease inhibitors (1:1000; GE Healthcare), RNAse and DNAse (1:1000; GE Healthcare), and 600-μm glass beads (1:1; Sigma). Cell debris and glass beads were removed via centrifugation at 5000 x *g* for 10 min, and dithiothreitol (final concentration 20 mM) was added to the supernatant [48]. Protein concentrations were determined using the Bradford method [49]. Cell extracts were kept at -80°C until use.

### Cat sera

Sera from cats with definitive diagnoses of sporotrichosis (via *S*. *brasiliensis* isolation in culture; n = 49) were obtained from INI/Fundação Oswaldo Cruz, Rio de Janeiro, Brazil. The distribution and number of skin lesions of the cats were classified as L1 (cutaneous lesion in only one place), L2 (cutaneous lesion in two non-adjacent places), or L3 (cutaneous lesions in three or more non-adjacent places). During examination, blood was collected via vein puncture and stored in an incubator for 1 h; serum was obtained via centrifugation and stored at -20°C until use. Sera from 19 cats with no evidence of sporotrichosis or other diseases (the control group) were obtained from São Paulo as described elsewhere [44]. Sera from 20 cats with other diseases were also studied to verify cross-reactions with feline infectious peritonitis/coronavirus (5 sera), feline leukemia virus (3 sera), feline immunodeficiency virus (2 sera), leptospirosis (3 sera), rickettsiosis (2 sera), erlichiosis (3 sera), and leishmaniasis (2 sera) as previously described [44] (S1 Diagram). All sera were stored at -20°C until use.

### ELISA

Sera from cats with confirmed sporotrichosis and from cats from the control group were tested via ELISA. To determine the best protein concentration for microplate sensitization, whole cellular proteins from *S*. *brasiliensis* (CBS 132990 and CBS 132021) and *S*. *schenckii s*. *str*. (CBS 132974 and CBS 132984) were tested and examined by checkerboard titration for antibody detection. Afterward, all microplates were sensitized with concentrations of 3.6μg/mL (100μL per well in 0.1 M carbonate-bicarbonate buffer, pH 9.6). High binding microtiter plates (Corning Costar, Corning) were sensitized for 2 h at 37°C and overnight at 4°C in a refrigerator. The remaining binding sites were blocked with phosphate-buffered saline containing 0.1% Tween 20 (PBST) and 5% non-fat dry milk (200 μL/well) for 4 h at 37°C. After washing three times with PBST, diluted serum (1:800 in PBST, 100μL/well) was added in duplicate for 1 h at 37°C. Afterward, 100μL horseradish peroxidase-conjugated goat anti-feline IgG (1:1000; Southern Biotech) were added to each well and incubated for 1 h at 37°C. After three washes with PBST, 100μL substrate solution (5 mg of o-phenylenediamine in 25 mL of 0.1 M citrate-phosphate buffer pH 5.0 plus 10 μL 30% H_2_O_2_) were added to each well, and the reaction was interrupted after 8 min in the dark by adding 50 μL 4 N H_2_SO_4_. Optical density was read at 492 nm with a Tecan Sunrise 96-well Microplate Reader (Tecan).

### Sodium dodecyl sulfate-polyacrylamide gel electrophoresis (SDS-PAGE) and 1D immunoblotting

*S*. *brasiliensis* and *S*. *schenckii* protein extracts (2 μg) were analyzed via SDS-PAGE with 10% gels [50] and silver-stained [51]. The relative molecular weights of the fractions were estimated using standard broad-range molecular weight markers (Protein Benchmark, Invitrogen).

For immunoblotting, proteins (10 μg) from strains CBS 132990, CBS 132021, CBS 132974, and CBS 132984 were resolved with SDS-PAGE and transferred onto 0.45-μm polyvinylidenedifluoride membranes (Bio-Rad) at 20 V for 30 min with transfer buffer (25 mM Tris base, 192 mM glycine, 20% methanol, pH 8.3) [52] using a Trans-Blot SD semi-dry device (Bio-Rad). Electrotransference was confirmed by staining with 0.15% Ponceau S and 1% acetic acid [vol/vol]. Membranes were destained and free binding sites were blocked overnight in phosphate-buffered saline blocking buffer (1% bovine serum albumin supplemented with 0.05% [vol/vol] Tween 20, 5% [wt/vol] skim milk, pH 7.6) at 4°C. To determine the best dilution of serum, one sample was tested at four dilutions (1:100, 1:200, 1:500, and 1:1000) against yeast extracts. Afterward, for all sera, membranes were probed individually with primary antibody diluted 1:500 at 25°C for 2 h. Membranes were washed three times with Tris-buffered saline (pH 7.5) containing 0.05% [vol/vol] Tween-20 for 10 min and incubated with horseradish peroxidase-conjugated goat anti-feline IgG (1:1000) for 2 h at room temperature. Membranes were then washed with Tris-buffered saline (pH 7.5) containing 0.05% [vol/vol] Tween-20 and signal was detected with an enhanced chemiluminescence detection kit (GE Healthcare). Blots were imaged in a transilluminator (Uvitec Cambridge). Allience 4.7 software was used to take several images at different time exposures, from 2 s each to a total of 10 images over 2 s.

### 2D gel electrophoresis and immunoblotting

Proteins were separated via 2D gel electrophoresis as previously described [45, 47]. Briefly, proteins (300 μg) were precipitated using the 2D Clean-up Kit (GE Healthcare) and resuspended in rehydration buffer (7 M urea, 2 M thiourea, 2% CHAPS, 1.2% DeStreak, 2% vol/vol isoelectric focusing buffer pH 4–7, and trace amounts of bromophenol blue) to a final volume of 250 μL. Immobilized pH gradient strips (pH 4–7, 13 cm; GE Healthcare) were rehydrated at 30 V for 12 h. Isoelectric focusing was performed at 20°C using a Multiphor III system (GE Healthcare) as follows: 200 V for 2 h, 500 V for 2 h, 1000 V for 5 h, and a gradient applied from 1000 to 5000 V for 2 h. Finally, the voltage was set to 5000 V for 60,000 Vhr. After 1D isoelectric focusing, the IPG strips were reduced for 15 min with 1.5% dithiothreitol and alkylated for 15 min with 2.5% iodocetamide in equilibration buffer (6 M urea, 50 mM Tris-HCl pH 6.8, 30% glycerol, and 2% sodium dodecyl sulfate). Second-dimension separation was carried out by placing equilibrated IPG strips onto 10 % polyacrylamide gels, sealing them with 0.5 % [wt/vol] low-melting-point agarose, and separating the proteins at 10°C using a Hoefer SE 600 unit (15 mA/gel for 30 min and then 23 mA/gel until the dye front reached the bottom of the gel). Proteins were developed with silver staining [51] or were directly transferred for immunoblotting.

For 2D immunoblotting, proteins were transferred onto 0.45-μm polyvinylidenedifluoride membranes at 25 V for 1 h with transfer buffer [52] using the Trans-Blot SD semi-dry system. The success of electrotransference was evaluated by staining with 0.15% Ponceau S and 1% acetic acid 1% [vol/vol]. Membranes were destained and free binding sites were blocked overnight in phosphate-buffered saline blocking buffer (1% bovine serum albumin supplemented with 0.05% [vol/vol] Tween 20, 5% [wt/vol] skim milk, pH 7.6) at 4°C.

Membranes obtained from 2D gels were probed with 1:500 primary antibody (gold standard pooled feline sera; n = 10) under the conditions used for 1D immunoblotting. Immunoreactive antigens were detected using an enhanced chemiluminescence detection kit (GE Healthcare). 2D immunoblots were imaged using the method used for 1D immunoblots.

### Statistical analysis

Diagnostic values included sensitivity, specificity, positive predictive value, and negative predictive value. Receiver operating characteristic (ROC) curves were prepared and analyzed to determine the sensitivity and specificity of each antigen preparation for ELISA. The area under the curve (AUC) for ROC analysis was calculated to evaluate the diagnostic value of ELISA. We assumed that a test lacked diagnostic power when the ROC curve was linear with an AUC of 0.5 (the ROC curve coincided with the diagonal). A powerful test was assumed to yield an AUC of ~1.0, indicating the absence of both false-positives and false-negatives (the ROC curve reached the upper left corner of the plot). To measure the degree of concordance of the results from preparations from strains CBS 132990, CBS 132021, CBS 132974, and CBS 132984, we calculated the kappa statistic and its 95% confidence interval (CI). Kappa values were interpreted as follows: 0.00–0.20, poor agreement; 0.21–0.40, fair agreement; 0.41–0.60, moderate agreement; 0.61–0.80, good agreement; 0.81–1.00, very good agreement [53]. *P*-values ≤0.05 were considered statistically significant. All calculations were performed with MedCalc Statistical Software version 14.8.1 (MedCalc Software bvba; http://www.medcalc.org). Findings are reported in line with the STARD checklist for studies of diagnostic accuracy (S1 Checklist).

## Results

**Fig 1:**
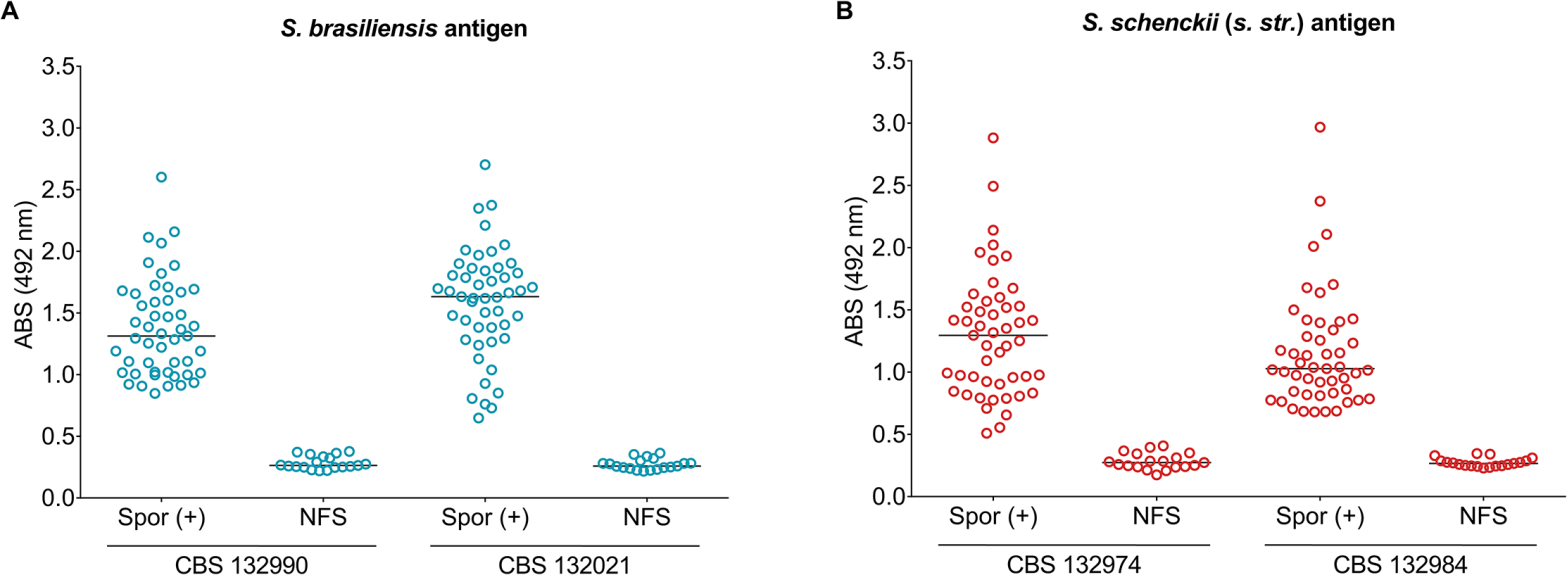
ELISA of antibody titers in sera from cats (n = 49) with confirmed sporotrichosis (Spor +). The range of antibody titers against *S*. *brasiliensis* (A) and *S*. *schenckii s*. *str*. (B) antigens were expressed as optical density (absorbance). Each symbol represents an individual cat; horizontal lines indicate the mean. Data are representative of two independent experiments. Titers from the control group (NFS: non-feline sporotrichosis) were significantly lower (*P*<0.001) than titers from infected cats (Spor +). Further information about ELISA statistics appears in S1 Table.

We previously reported a high prevalence of *S*. *brasiliensis* in feline sporotrichosis outbreaks [8, 9, 20]. Based on this information, the main goal of the present investigation was to evaluate the presence and diversity of serum-derived antibodies against *S*. *brasiliensis* antigens in naturally infected cats. Further, we previously proposed the existence of a convergent humoral response in human sporotrichosis against antigens from *S*. *brasiliensis*, *S*. *schenckii*, and *S*. *globosa* [45]. To establish whether *S*. *brasiliensis* and *S*. *schenckii* express different antigens, we assessed whole cellular protein extracts from two strains of *S*. *brasiliensis* plus two strains of *S*. *schenckii s*. *str*. that were previously characterized by molecular [8, 9, 25, 54] and serological [3, 44–47] methods. Remarkably, and in support of our hypothesis that immunological distance increases with phylogenetic distance, sera from these cats reacted similarly, with no significant differences in titer between ELISA plates coated with proteins from *S*. *brasiliensis* or *S*. *schenckii* (Fig 1).

**Fig 2:**
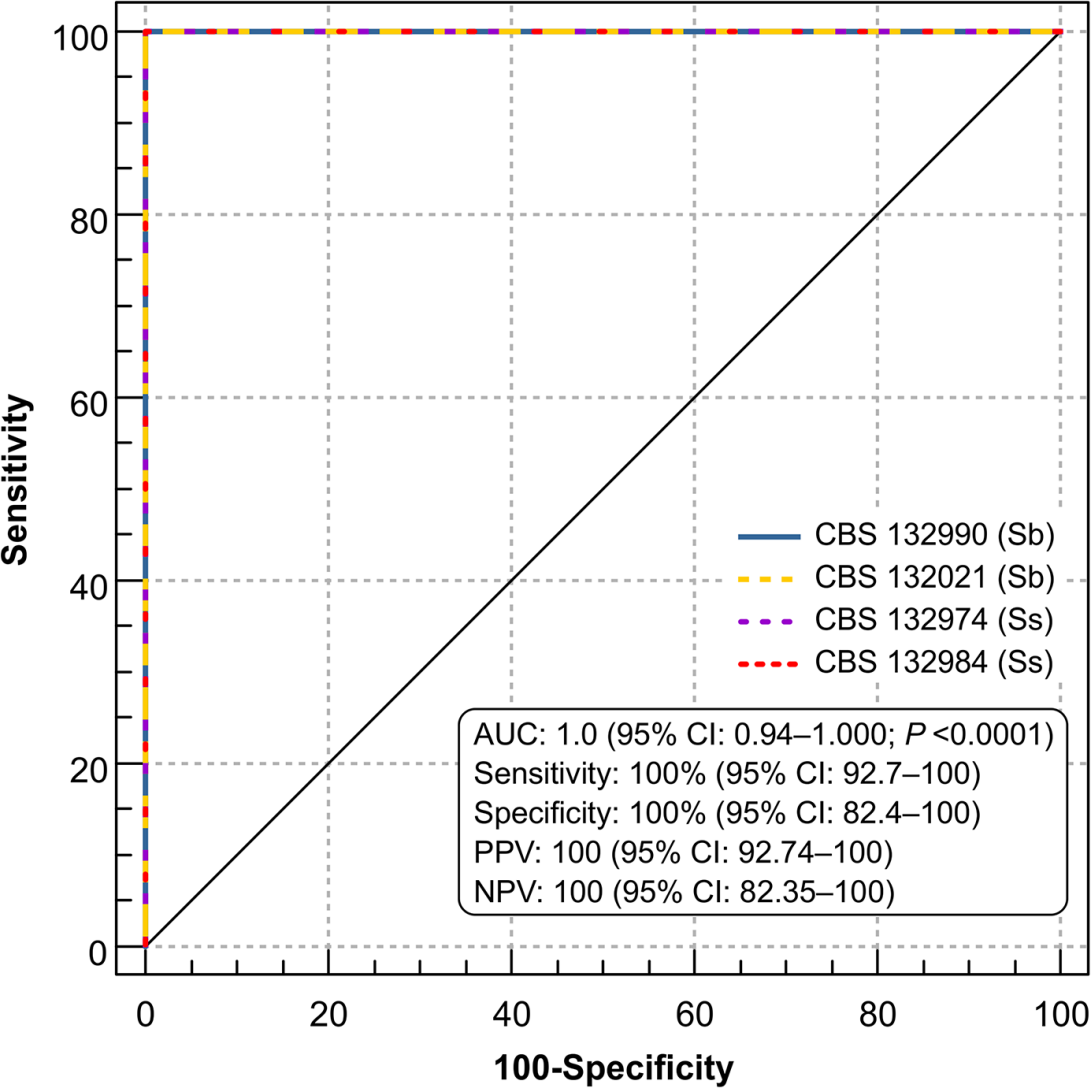
ROC analysis of assay sensitivity and specificity. Samples consisted of sera from 49 infected cats and 19 uninfected cats. Sb, *S*. *brasiliensis*; Ss, *S*. *schenckii*; PPV, positive predictive value; NPV, negative predictive value.

ELISA detection of the four antigen preparations exhibited similar results, medians, and ranges for cats infected with sporotrichosis (n = 49): *S*. *brasiliensis* CBS 132990, median 1.313 OD, 95% CI 1.262–1.489 OD; *S*. *brasiliensis* CBS 132021, median 1.632 OD, 95% CI 1.462–1.714 OD; *S*. *schenckii* CBS 132974, median 1.296 OD, 95% CI 1.157–1.442 OD; and *S*. *schenckii* CBS 132984, median 1.028 OD, 95% CI 1.027–1.294 OD (S1 Table). When using the assay to diagnosis cats with sporotrichosis, the area under the ROC curve was 1.0 (95% CI 0.94–1.000; *P*<0.0001; Fig 2), indicating excellent performance. The control group of 19 non-*Sporothrix* infected animals was associated with lower medians and smaller ranges: *S*. *brasiliensis* CBS 132990, median 0.2640 OD, 95% CI 0.2592–0.3098 OD; *S*. *brasiliensis* CBS 132021, median 0.2590 OD, 95% CI 0.2517–0.2942 OD; *S*. *schenckii* CBS 132974, median 0.2730 OD, 95% CI 0.2512–0.3136 OD; and *S*. *schenckii* CBS 132984, median 0.2670 OD, 95% CI 0.2567–0.2907 OD (S1 Table). Differences between the absorbance values for the infected and non-infected groups were statistically significant (*P*<0.0001). Sera from cats with other infections were non-reactive. Similar cutoff values yielded 100% specificity and sensitivity: *S*. *brasiliensis* CBS 132990, 0.377 OD; *S*. *brasiliensis* CBS 132021, 0.363 OD; *S*. *schenckii* CBS 132974, 0.407 OD; and *S*. *schenckii* CBS 132984, 0.346 OD (S1 Fig). ELISA results showed very good agreement for the antigens assayed (kappa = 1.0). To diagnosis feline sporotrichosis via ELISA, we recommend the use of antigen preparations of *S*. *brasiliensis*, since this is the most prevalent species in feline sporotrichosis outbreaks.

Antigen diversity was assayed with 1D immunoblots using the four antigen preparations of *S*. *brasiliensis* and *S*. *schenckii* tested via ELISA. Proteins extracts were evaluated according to the amount of protein extracted, the diversity of bands, the integrity of the samples, and the reproducibility of extraction. Approximately 2 μg of *Sporothrix* yeast whole-cell extracts were resolved by SDS-PAGE; silver staining revealed numerous proteins ranging from 10 kDa to 160 kDa in size, with different intensities. The Tris-Ca^2+^ extraction protocol [45, 47] was suitable for the study of *Sporothrix* antigenic molecules during feline sporotrichosis, yielding samples with high amounts of protein and no degradation.

**Fig 3:**
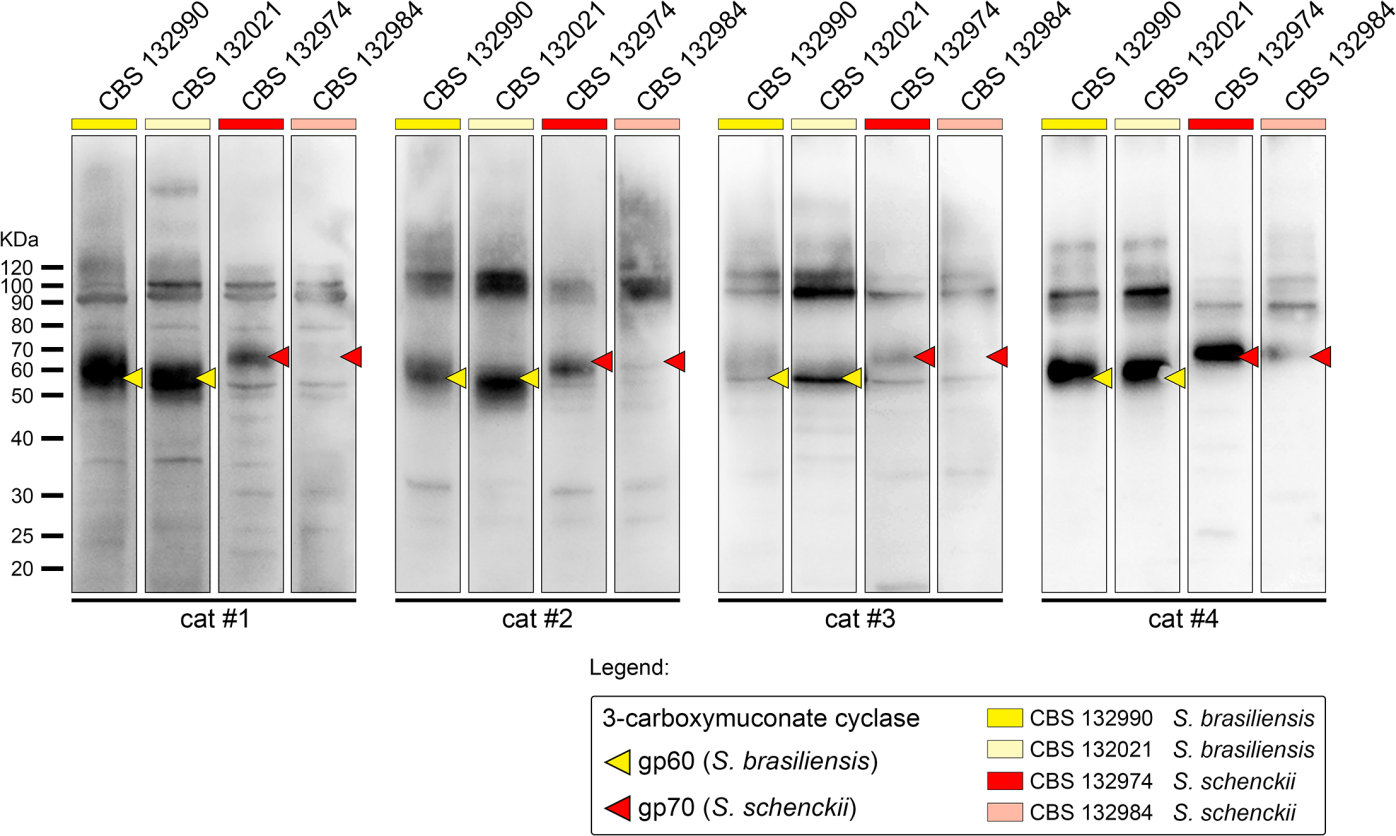
Representative immunoblot of *S*. *brasiliensis* (CBS 132990 and CBS 132021) and *S*. *schenckii* (CBS 132974 and CBS 132984) antigens using sera from cats with confirmed sporotrichosis. Despite the diversity of recognition patterns in different animals, the humoral response was consistently strong against the immunodominant antigen 3-carboxymuconate cyclase (gp60 in *S*. *brasiliensis* and gp70 in *S*. *schenckii s*. *str*.). Immunoblotting is as described in Methods.

As expected, antibodies from cats with sporotrichosis reacted with a wide variety of *S*. *brasiliensis* and *S*. *schenckii* proteins 20kDa to >160kDa in size (Fig 3). Cat-to-cat variation resulted in characteristic banding patterns for each animal (Fig 3); supporting the hypothesis that in a genetically diverse population, the antibody repertoire is expected to vary among individual cats. On the other hand, we detected minor or no differences in IgG-reacting banding patterns between antigen preparations (Fig 3), consistent with the close genetic distance between *S*. *brasiliensis* and *S*. *schenckii* [8, 9]. Despite this variation, all cats produced antibodies against a 60-kDa molecule in the *S*. *brasiliensis* proteome and a 70-kDa molecule in the *S*. *schenckii* proteome.

**Fig 4:**
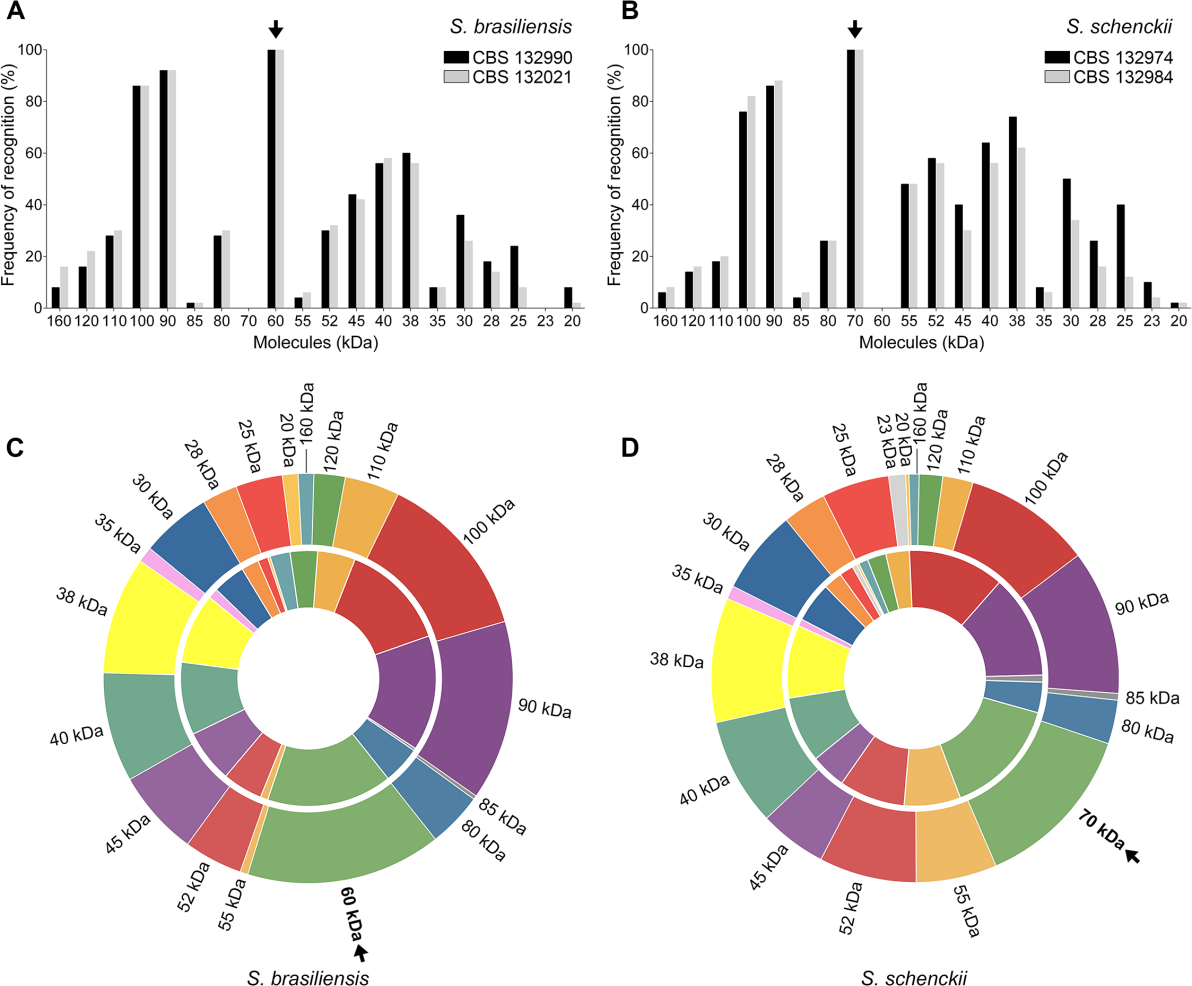
Frequency and diversity of serum-derived antibodies (IgG isotype) against *S*. *brasiliensis* and *S*. *schenckii* antigens in naturally infected cats. 3-carboxymuconate cyclase (gp60 in the *S*. *brasiliensis* proteome (A); gp70 in the *S*. *schenckii* proteome (B)) is the immunodominant molecule in feline sporotrichosis; it was recognized by 100% of the cat sera tested here irrespective of disease form and severity. (C) Diversity of recognition of *S*. *brasiliensis* antigens (outer ring, CBS 132990; inner ring, CBS 132021). (D) Diversity of recognition of *S*. *schenckii* antigens (outer ring, CBS 132974; inner ring, CBS 132984). Charts are proportional. Molecular weights are colored as indicated. Further information about the frequency of antigen recognition appears in S2 Table.

The major antigenic *S*. *brasiliensis* molecules (CBS 132990 and CBS 132021) recognized by feline IgG consisted of the following sizes: 60 kDa (100% and 100%, respectively), 90 kDa (92% and 92%, respectively), 100 kDa (86% and 86%, respectively), 38 kDa (60% and 56%, respectively), 40 kDa (56% and 58%, respectively), 45 kDa (44% and 42%, respectively), 30 kDa (36% and 26%, respectively), 52 kDa (30% and 32%, respectively), and 110 kDa (28% and 30%, respectively) (Fig 4A and 4C). Minor molecules recognized by feline IgG had sizes of 80 kDa, 25 kDa, 28 kDa, 120 kDa, 160 kDa, 35 kDa, 20 kDa, 55 kDa, 85 kDa, and 23 kDa (Fig 4A and 4C). The major antigenic *S*. *schenckii* molecules (CBS 132974 and CBS 132984) recognized by feline IgG had sizes of: 70 kDa (100% and 100%, respectively), 90 kDa (86% and 88%, respectively), 100 kDa (76% and 82%, respectively), 38 kDa (74% and 62%, respectively), 40 kDa (64% and 56%, respectively), 52 kDa (58% and 56%, respectively), 30 kDa (50% and 34%, respectively), 55 kDa (48% and 48%, respectively), and 45 kDa (40% and 30%, respectively) (Fig 4B and 4D). The minor *S*. *schenckii* molecules recognized by feline IgG had sizes of 25 kDa, 80 kDa, 28 kDa, 110 kDa, 120 kDa, 23 kDa, 35 kDa, 160 kDa, 85 kDa, and 20 kDa (Fig 4B and 4D). Sera from uninfected cats did not react with *S*. *brasiliensis* or *S*. *schenckii* antigens. Sera from cats with other infections were also non-reactive in the immunoblot assay. The frequencies at which *Sporothrix* molecules were recognized in the antigen preparations are presented in S2 Table. There was no association between the number of bands recognized by each serum and the distribution and number of skin lesions on cats with sporotrichosis.

**Fig 5:**
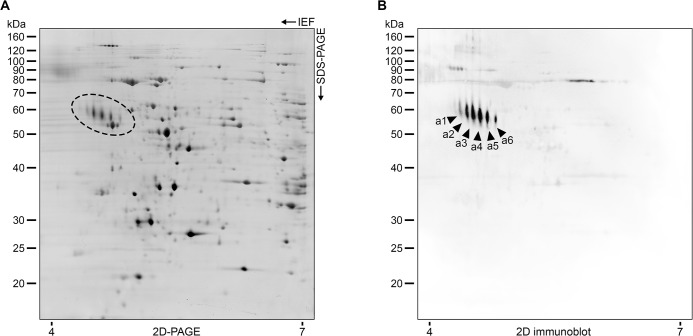
Identification of IgG-reactive proteins from the *S*. *brasiliensis* proteome via 2D immunoblotting. (A) Proteins from isolate CBS 132990 were fractionated using 13 cm pH 4–7 strips (left to right) in the first dimension and 10% SDS-PAGE in the second dimension. (B) Immunoproteomics of *S*. *brasiliensis* during feline sporotrichosis. Serum-derived antibodies (pooled sera; n = 10) in naturally infected cats mainly recognized all six isoforms of 3-carboxymuconate cyclase (gp60) in the *S*. *brasiliensis* proteome (a1-a6; GenBank: KP233225). Immunoblotting is as described in Methods.

We previously reported that the 60-kDa and 70-kDa proteins in *S*. *brasiliensis* and *S*. *schenckii*, respectively, are related to virulence profiles and are the main antigenic molecules during murine [3] and human [45] sporotrichosis. We also determined that this protein undergoes post-translational modification and is present as isoforms and glycoforms in the *S*. *brasiliensis* and *S*. *schenckii* proteomes [45]. We therefore investigated whether antibodies present in cat sera recognize all six isoforms in the *S*. *brasiliensis* proteome, as previously shown with human antibodies [45]. *S*. *brasiliensis* proteins were therefore resolved via 2D electrophoresis and immunoblotted with pooled sera from cats with sporotrichosis (n = 10) and optimal antibody titers according to ELISA. Serum-derived antibodies in naturally infected cats mainly recognized all six isoforms of gp60 (Fig 5). The present results confirm that *S*. *brasiliensis* 3-carboxymuconate cyclase is a highly polymorphic protein [45] with sizes of 55–62 kDa and with isoelectric points of 4.45–4.80. 2D immunoblotting revealed less diversity and more weakly reacting spots than 1D immunoblotting, perhaps due to protein loss during sample preparation for 2D electrophoresis (compared to the crude extracts used in 1D immunoblotting and ELISA) and serum dilution during pooling.

## Discussion

In the past, sporotrichosis was reported in horses more frequently than in other animals [55, 56]. Although the disease has been reported in several animal species, cats are currently the most frequently affected domestic animal. Outdoor cats are exposed to the fungus through contact with natural environmental sources or other sick cats. Cats have gained importance in the zoonotic transmission of *Sporothrix* to humans [8, 28–30, 57, 58]. Presently, the all-time high number of feline sporotrichosis cases reported in Brazil has reached epidemic proportions [8, 20, 29, 59]. Unlike humans, cats are highly susceptible to this fungus due to the low frequency of granulomas and the richness of fungal elements observed during skin histopathology [18]. Moreover, unlike human-disseminated sporotrichosis, which classically affects immunocompromised individuals [24, 26], systemic disease in cats occurs frequently and is not associated with immunodeficiency caused by feline immunodeficiency virus and/or feline leukemia virus [18]. In this scenario, absence of an efficient host immune response is a key factor in disease progression. The low frequency of granulomas and uncontrolled fungal growth suggest that a lack of adequate cellular immunity underlies disease severity and pathology.

ELISA has been achieved with various antigen preparations, thus enabling serodiagnosis of human [41–43, 60, 61] and feline [44] sporotrichosis. The present investigation suggests that ELISA-based quantitation of anti-*S*. *brasiliensis* IgG is remarkably sensitive for the detection of feline sporotrichosis (cutoffs of 0.346–0.407 OD); as expected, this strategy does not differentiate between *S*. *brasiliensis* or *S*. *schenckii* as the agent of infection. Despite its high prevalence in South and Southeast Brazil, *S*. *brasiliensis* infection in cats is not an exclusive host-pathogen association, since the sibling agent *S*. *schenckii s*. *str*. also occurs in cats in Brazil [8, 9, 20] and Malaysia [62], albeit with significantly lower frequency. Our serology-based observations of a convergent antigenic response are similar to previous 2D immunoblotting results for human sporotrichosis [45]. Interestingly, in other thermally dimorphic fungi such as *Paracoccidioides brasiliensis* and *P*. *lutzii* (Onygenales), antigen composition seems to vary considerably between species, an observation that supported development of a differential diagnosis system based on titration of serum-derived antibodies from humans infected with distinct strains of *Paracoccidioides* [63]. It is likely that similarities in antigen profiling among clinical *Sporothrix* spp. (*S*. *brasiliensis*, *S*. *schenckii*, and *S*. *globosa*) could be related to a recent speciation event and therefore be less susceptible to variability; however, this hypothesis requires further investigation. In addition, factors related to environment or to host association may impose strong selection pressures on *Sporothrix* antigen profiles.

To date, no information about the humoral response in feline sporotrichosis has been reported in the literature; it is a completely unknown area in veterinary medicine that merits exploration. Here, sera from cats with sporotrichosis displayed immunoblotting patterns of *Sporothrix*-specific immunogenic molecules that were markedly different from patterns from sera from uninfected cats. These immunogenic components were 20–160 kDa in molecular weight. The main molecules recognized by cat sera were a 60-kDa protein in the *S*. *brasiliensis* proteome and a 70-kDa protein in *S*. *schenckii s*. *str*., followed by molecules weighing 100 kDa, 90 kDa, 40 kDa, and 38 kDa. The variety of antigenic components recognized by each serum may be due to specific antigens secreted by individual fungal strains [3, 64] as well as to different mechanisms of activation of each host’s immune system [45, 65]. However, variation in antibody repertoire seems reasonable in a genetically diverse host population [66].

We interpreted published data lacking taxonomic information or protein identification via matrix-assisted laser desorption/ionization time of flight (MALDI-ToF)/mass spectrometry (MS) and identified an immunodominant fraction that oscillated between 60 kDa and 70 kDa in various studies (Table 2). In this scenario, the humoral immune response may be influenced by the infection strain and the antigen preparation used to detect the humoral response. Regarding human sporotrichosis, Mendoza et al. [67] observed that 147-kDa, 90-kDa, 74-kDa, 55-kDa, and 40-kDa molecules are commonly recognized by sera from patients with sporotrichosis, and Scott & Muchmore [68] showed that 70-kDa, 40-kDa, 36-kDa, and 22-kDa molecules are immunodominant in human sporotrichosis. A 70-kDa molecule was previously highlighted as immunodominant during murine sporotrichosis [69–71], with IgG1 and IgG3 predominant [71]. This 70-kDa molecule was originally described as an adhesin molecule for fibronectin and laminin in *S*. *schenckii s*. *str*. [69, 70] that localized to the fungal cell wall [46]. More recently, we identified this molecule via 2D immunoblotting followed by MALDI-ToF/MS as 3-carboxymuconate cyclase (GenBank: KP233225), the major antigen of human sporotrichosis [45]. Based on 1D electrophoresis [3, 46] and 2D electrophoresis [45], the molecular weight (55–73kDa) and isoelectric point (4.33–4.85) of 3-carboxymuconate cyclase vary intra- and interspecifically [3, 45, 46, 64]. Variation in gp60/gp70 may be related to differential glycosylation patterns and amino-acid substitution along the protein core, since all glycoforms/isoforms display identical MALDI-ToF/MS spectra [45] (Table 2).

**Table 2:** Summary of studies reporting humoral immune responses against the major *Sporothrix* spp. antigenic molecules.

Study / Reference	Species / Antigen	Major molecule	Immunoblot	Serum source	MS	Genbank
Scott & Muchmore [68]	*S*. *schenckii s*. *l*.	70-kDa	1D	Human	No	-
Mendoza et al. [67]	*S*. *schenckii s*. *l*.	74-kDa	1D	Human	No	-
Carlos et al. [72]	*S*. *schenckii s*. *l*.	67-kDa	1D	Murine	No	-
Nascimento & Almeida [71]	*S*. *schenckii s*. *str*.	70-kDa	1D	Murine	No	-
Ruiz-Baca et al. [73]	*S*. *schenckii s*. *l*.	70-kDa	1D/2D	Rabbit	No	-
Fernandes et al. [3]	*S*. *brasiliensis*	60-kDa	1D	Murine	No	-
	*S*. *schenckii s*. *str*.	70-kDa	1D	Murine	No	-
Castro et al. [46]	*S*. *brasiliensis*	60-kDa	1D	Murine	Yes	KF275146
	*S*. *schenckii s*. *str*.	70-kDa	1D	Murine	Yes	KF275147
Ruiz-Baca et al. [74]	*S*. *brasiliensis*	60-kDa	2D	Rabbit	No	-
	*S*. *schenckii s*. *str*.	70-kDa	2D	Rabbit	No	-
Rodrigues et al. [45]	*S*. *brasiliensis*	60-kDa	2D	Human	Yes	KP233225
	*S*. *schenckii s*. *str*.	70-kDa	2D	Human	Yes	KP233225
	*S*. *globosa*	70-kDa	2D	Human	Yes	KP233225
Rodrigues et al.	*S*. *brasiliensis*	60-kDa	1D/2D	Feline	Yes	KP233225
(this study)	*S*. *schenckii s*. *str*.	70-kDa	1D	Feline	Yes	KP233225

*s*.*l*.: *sensu lato*; *s*. *str*.: *sensu stricto*; 1D: one-dimensional; 2D: two-dimensional; MS: mass spectrometry.

Immunogenic proteins oscillating between 60- and 70-kDa were reported in the literature and identified by MS as 3-carboxymuconate cyclase, in human, murine and feline sporotrichosis.

Here, the intensity of the recognition of distinct molecules differed among sera from different cats, but serum from an individual cat displayed little variation when probing different antigen preparations. These data suggest that the antibody response differs between cats and that there are few qualitative variations in the expression of cellular antigens by *S*. *brasiliensis* and *S*. *schenckii s*. *str*. In this study, the clinical presentation of sporotrichosis in cats corresponded mainly to multiple skin lesions; we observed no association between the distribution and number of skins lesions and the number or type of molecules recognized by antibodies in the sera. Although it remains to be clarified whether the antibodies produced during active infection in feline sporotrichosis are protective, antibodies against 3-carboxymuconate cyclase appear to inhibit fungal adhesion to the host in a dose-dependent manner [70, 73]. In another dimorphic fungus, *P*. *brasiliensis*, passive administration of monoclonal antibodies against the immunodominant antigen gp43 or against the recombinant protein before and after intratracheal or intravenous infections reduced fungal burden and decreased pulmonary inflammation in mice [75, 76].

### Conclusions

Gaining insight into host-parasite interplay in the immunological context is essential for understanding the emergence of feline sporotrichosis and is critical to serodiagnosis and the development of vaccines. Here, we demonstrated that antigens derived from yeast cell extracts of *S*. *brasiliensis* and *S*. *schenckii s*. *str*. yielded excellent results in ELISA and immunoblotting. The variety of molecules recognized by sera may be related to certain characteristics of the isolate, such as virulence, or even related to immune-system activation in each individual host. During infection, *Sporothrix* antigens elicit an IgG-mediated response; 3-carboxymuconate cyclase (gp60 in *S*. *brasiliensis* and gp70 in *S*. *schenckii*) is the immunodominant molecule in feline sporotrichosis, similar to murine and human disease. Therefore, this molecule may also be useful as a marker in the diagnosis of feline sporotrichosis and is a promising candidate for the development of therapeutic vaccines to tackle sporotrichosis in highly endemic areas.

## Supporting Information

S1 Checklist STARD checklist. (DOC)

S1 Table Summary statistics for antigen detection via ELISA with four antigenic extracts. (DOC)

S2 Table Frequencies of antigen recognition on immunoblotting with serum-derived antibodies against *S*. *brasiliensis* and *S*. *schenckii* proteins in naturally infected cats (n = 49). (DOC)

S1 Fig ELISA with *S*. *brasiliensis* and *S*. *schenckii* yeast cell extracts holds great potential for diagnosing feline sporotrichosis. ELISA-based quantitation of IgG against *Sporothrix* antigens has remarkable high sensitivity (Sens) and specificity (Spec) in infected (diagnosis = 1) and non-infected (diagnosis = 0) animals. Similar cutoff values yielded 100% specificity and sensitivity: (A) *S*. *brasiliensis* (Sb) CBS 132990, 0.377 OD; (B) *S*. *brasiliensis* CBS 132021, 0.363 OD; (C) *S*. *schenckii* (Ss) CBS 132974, 0.407 OD; and (D) *S*. *schenckii* CBS 132984, 0.346 OD.
(TIF)

S1 Diagram STARD flow diagram. (PDF)
