# Supplementary material for: Proteomics-Based Characterization of the Humoral Immune Response in Sporotrichosis: Toward Discovery of Potential Diagnostic and Vaccine Antigens
Source: PLoS Negl Trop Dis. 2015 Aug 25;9(8):e0004016. doi: 10.1371/journal.pntd.0004016 (PMC4549111; doi:10.1371/journal.pntd.0004016)
Supplement: S1 Table — (DOC) [file pntd.0004016.s002.doc]

**Table S1. Summary statistics for antigen detection via ELISA with four antigenic extracts.**

| Species/Antigen* | *Sporothrix brasiliensis* | | | | *Sporothrix schenckii s. str.* | | | |
| --- | --- | --- | --- | --- | --- | --- | --- | --- |
|  | CBS 132990 | | CBS 132021 | | CBS 132974 | | CBS 132984 | |
| Group | Spor (+) ^a^ | NFS ^b^ | Spor (+) ^a^ | NFS ^b^ | Spor (+) ^a^ | NFS ^b^ | Spor (+) ^a^ | NFS ^b^ |
| Number of serum | 49 | 19 | 49 | 19 | 49 | 19 | 49 | 19 |
| Median | 1.313 | 0.264 | 1.632 | 0.259 | 1.296 | 0.273 | 1.028 | 0.267 |
| Minimum | 0.848 | 0.22 | 0.648 | 0.217 | 0.509 | 0.175 | 0.68 | 0.229 |
| Maximum | 2.602 | 0.377 | 2.702 | 0.363 | 2.881 | 0.407 | 2.967 | 0.346 |
| 25% Percentile | 1.017 | 0.249 | 1.338 | 0.239 | 0.9135 | 0.238 | 0.825 | 0.247 |
| 75% Percentile | 1.662 | 0.335 | 1.852 | 0.302 | 1.549 | 0.344 | 1.368 | 0.287 |
| Mean | 1.376 | 0.2845 | 1.588 | 0.2729 | 1.299 | 0.2824 | 1.161 | 0.2737 |
| Std. Deviation | 0.3947 | 0.05245 | 0.4383 | 0.04413 | 0.4972 | 0.06479 | 0.4649 | 0.03526 |
| Std. Error of Mean | 0.05639 | 0.01203 | 0.06261 | 0.01012 | 0.07102 | 0.01486 | 0.06641 | 0.008088 |
| Lower 95% CI of mean | 1.262 | 0.2592 | 1.462 | 0.2517 | 1.157 | 0.2512 | 1.027 | 0.2567 |
| Upper 95% CI of mean | 1.489 | 0.3098 | 1.714 | 0.2942 | 1.442 | 0.3136 | 1.294 | 0.2907 |

*Values are expressed as optical density (O.D.);

^a^ Spor (+): Positive for *Sporothrix.* Group composed of naturally infected animals, with *S. brasiliensis* culture proven;

^b^ NFS: normal feline serum. Group composed of non-infected animals.
