# Supplementary material for: Proteomics-Based Characterization of the Humoral Immune Response in Sporotrichosis: Toward Discovery of Potential Diagnostic and Vaccine Antigens
Source: PLoS Negl Trop Dis. 2015 Aug 25;9(8):e0004016. doi: 10.1371/journal.pntd.0004016 (PMC4549111; doi:10.1371/journal.pntd.0004016)
Supplement: S2 Table — (DOC) [file pntd.0004016.s003.doc]

**Table S2. Frequencies of antigen recognition on immunoblotting with serum-derived antibodies against *S. brasiliensis* and *S. schenckii* proteins in naturally infected cats (n=49).**

| Molecule (kDa) | *S. brasiliensis* | | *S. schenckii* | |
| --- | --- | --- | --- | --- |
|  | CBS 132990 | CBS 132021 | CBS 132974 | CBS 132984 |
| 160 | 8 | 16 | 6 | 8 |
| 120 | 16 | 22 | 14 | 16 |
| 110 | 28 | 30 | 18 | 20 |
| 100 | 86 | 86 | 76 | 82 |
| 90 | 92 | 92 | 86 | 88 |
| 85 | 2 | 2 | 4 | 6 |
| 80 | 28 | 30 | 26 | 26 |
| **70** | 0 | 0 | **100** | **100** |
| **60** | **100** | **100** | 0 | 0 |
| 55 | 4 | 6 | 48 | 48 |
| 52 | 30 | 32 | 58 | 56 |
| 45 | 44 | 42 | 40 | 30 |
| 40 | 56 | 58 | 64 | 56 |
| 38 | 60 | 56 | 74 | 62 |
| 35 | 8 | 8 | 8 | 6 |
| 30 | 36 | 26 | 50 | 34 |
| 28 | 18 | 14 | 26 | 16 |
| 25 | 24 | 8 | 40 | 12 |
| 23 | 0 | 0 | 10 | 4 |
| 20 | 8 | 2 | 2 | 2 |

Data are %.

Gp60 in the *S. brasiliensis* proteome and gp70 in the *S. schenckii* proteome were highlighted in yellow as it was found to be the immunodominant antigen in feline sporotrichosis. This protein was identified earlier by MALDI-ToF MS/MS as 3-caboxymuconate cyclase (GenBank accession number: KP233225), the major antigen in human sporotrichosis (Rodrigues *et al.*, 2015).

**Supplementary Reference**

Rodrigues AM, Kubitschek-Barreira PH, Fernandes GF, de Almeida SR, Lopes-Bezerra LM, de Camargo ZP. Immunoproteomic analysis reveals a convergent humoral response signature in the *Sporothrix schenckii* complex. J Proteomics. 2015;115:8-22.
