# Supplementary figures and images for: Proteomics-Based Characterization of the Humoral Immune Response in Sporotrichosis: Toward Discovery of Potential Diagnostic and Vaccine Antigens
Source: PLoS Negl Trop Dis. 2015 Aug 25;9(8):e0004016. doi: 10.1371/journal.pntd.0004016 (PMC4549111; doi:10.1371/journal.pntd.0004016)

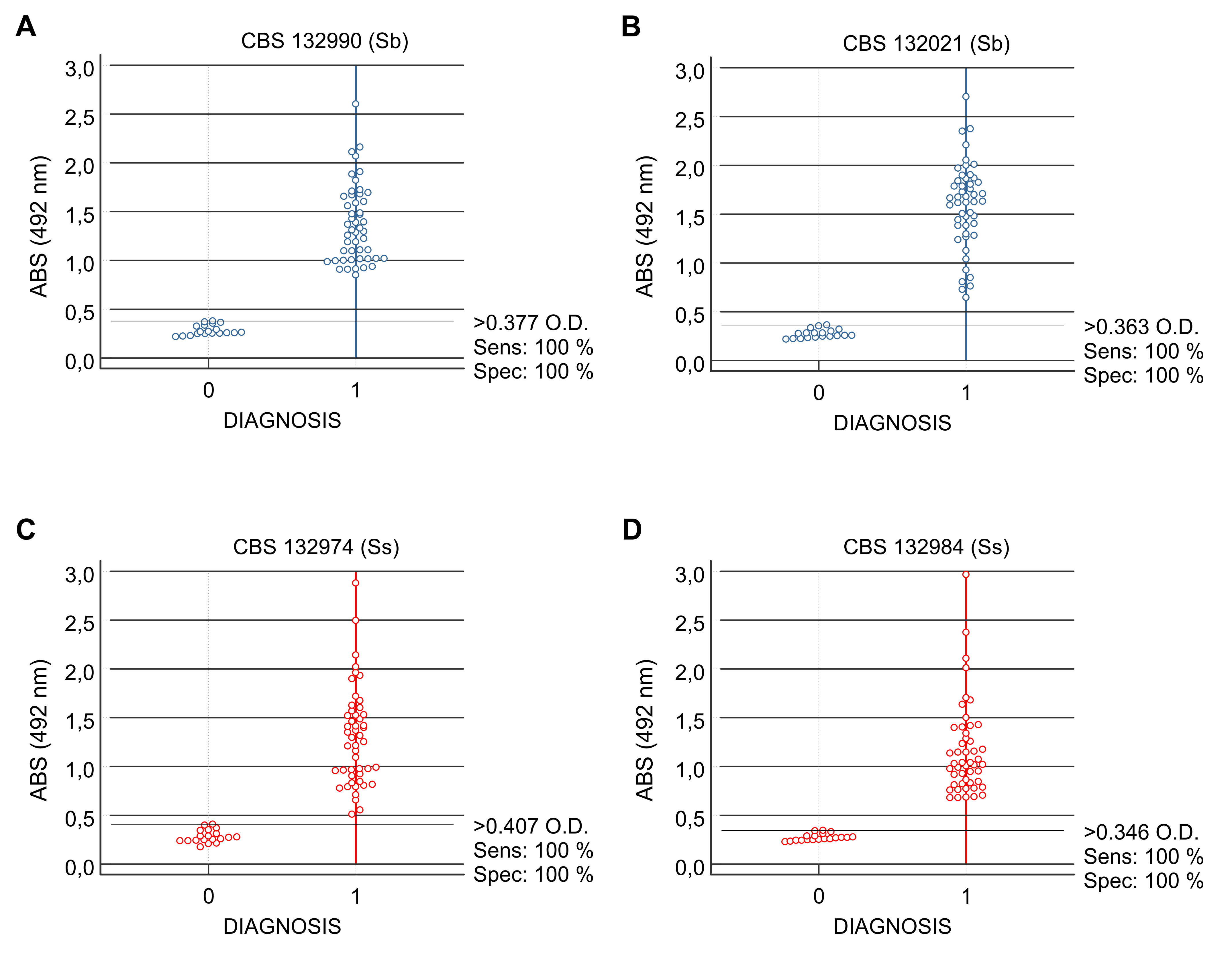

Supplement: S1 Fig — ELISA-based quantitation of IgG against Sporothrix antigens has remarkable high sensitivity (Sens) and specificity (Spec) in infected (diagnosis = 1) and non-infected (diagnosis = 0) animals. Similar cutoff values yielded 100% specificity and sensitivity: (A) S. brasiliensis (Sb) CBS 132990, 0.377 OD; (B) S. brasiliensis CBS 132021, 0.363 OD; (C) S. schenckii (Ss) CBS 132974, 0.407 OD; and (D) S. schenckii CBS 132984, 0.346 OD. (TIF) [file pntd.0004016.s004.tif]

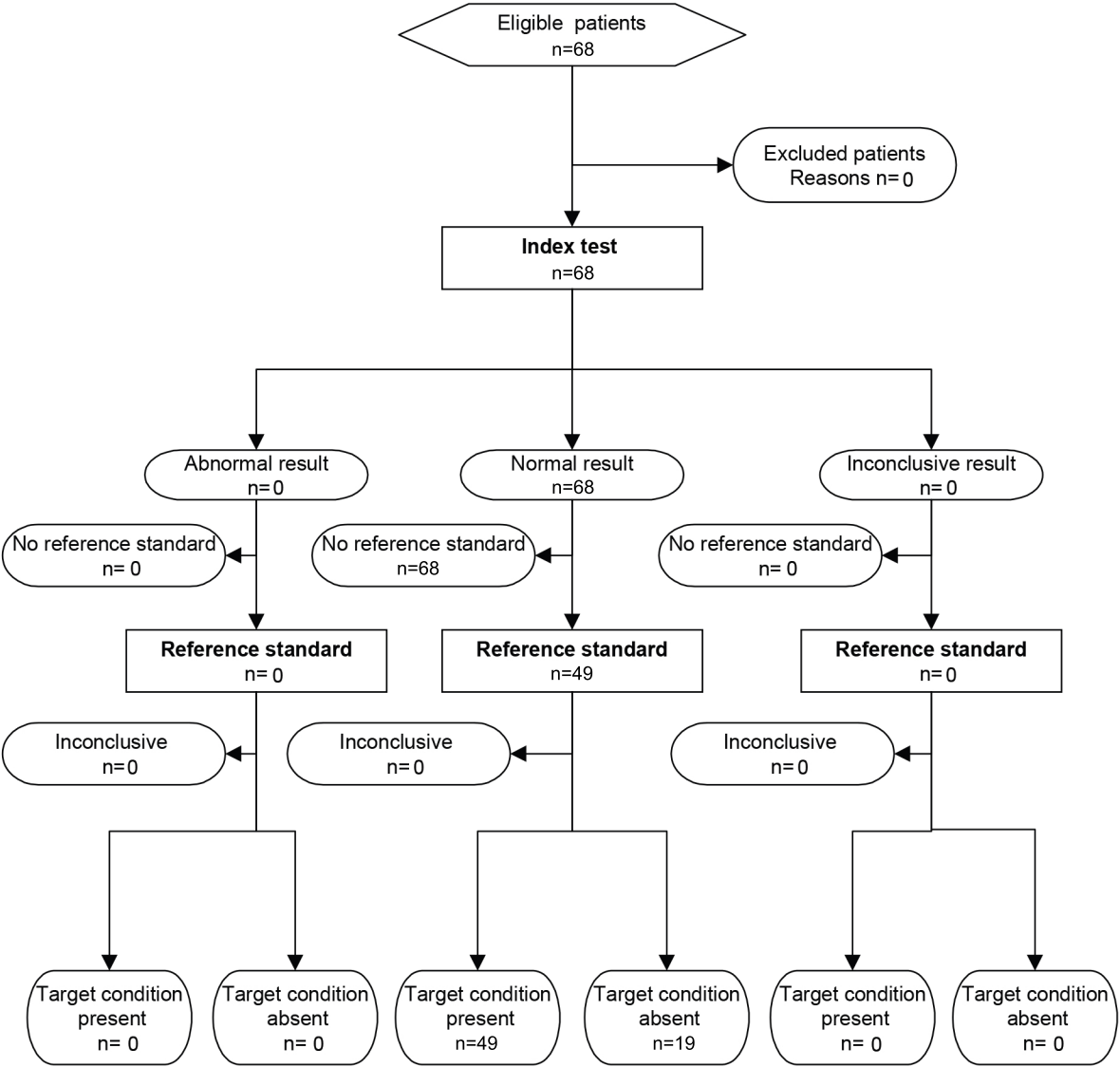

Supplement: S1 Diagram — (PDF) [file pntd.0004016.s005.pdf]
